# Inhibition of CXCR1/2 reduces the emperipolesis between neutrophils and megakaryocytes in the *Gata1*^low^ model of myelofibrosis

**DOI:** 10.1016/j.exphem.2023.02.003

**Published:** 2023-03-01

**Authors:** Francesca Arciprete, Paola Verachi, Fabrizio Martelli, Mauro Valeri, Manjola Balliu, Paola Guglielmelli, Alessandro Maria Vannucchi, Anna Rita Migliaccio, Maria Zingariello

**Affiliations:** a Unit of Microscopic and Ultrastructural Anatomy, University Campus Bio-Medico, Rome, Italy;; b Department of Biomedical and Neuromotor Sciences, University of Bologna, Bologna, Italy;; c National Center for Preclinical and Clinical Research and Evaluation of Pharmaceutical Drugs, Istituto Superiore di Sanità, Rome, Italy;; d Center for Animal Experimentation and Well-Being, Istituto Superiore di Sanità, Rome, Italy;; e Center Research and Innovation of Myeloproliferative Neoplasm, University Hospital Careggi, University of Florence, Florence, Italy;; f Altius Institute for Biomedical Sciences, Seattle, WA

## Abstract

Emperipolesis between neutrophils and megakaryocytes was first identified by transmission electron microscopy. Although rare under steady-state conditions, its frequency greatly increases in myelofibrosis, the most severe of myeloproliferative neoplasms, in which it is believed to contribute to increasing the transforming growth factor (TGF)-*β* microenvironmental bioavailability responsible for fibrosis. To date, the challenge of performing studies by transmission electron microscopy has hampered the study of factors that drive the pathological emperipolesis observed in myelofibrosis. We established a user-friendly confocal microscopy method that detects emperipolesis by staining with CD42b, specifically expressed on megakaryocytes, coupled with antibodies that recognize the neutrophils (Ly6b or neutrophil elastase antibody). With such an approach, we first confirmed that the bone marrow from patients with myelofibrosis and from *Gata1*^low^ mice, a model of myelofibrosis, contains great numbers of neutrophils and megakaryocytes in emperipolesis. Both in patients and *Gata1*^low^ mice, the emperipolesed megakaryocytes were surrounded by high numbers of neutrophils, suggesting that neutrophil chemotaxis precedes the actual emperipolesis event. Because neutrophil chemotaxis is driven by CXCL1, the murine equivalent of human interleukin 8 that is expressed at high levels by malignant megakaryocytes, we tested the hypothesis that neutrophil/megakaryocyte emperipolesis could be reduced by reparixin, an inhibitor of CXCR1/CXCR2. Indeed, the treatment greatly reduced both neutrophil chemotaxis and their emperipolesis with the megakaryocytes in treated mice. Because treatment with reparixin was previously reported to reduce both TGF-*β* content and marrow fibrosis, these results identify neutrophil/megakaryocyte emperipolesis as the cellular interaction that links interleukin 8 to TGF-*β* abnormalities in the pathobiology of marrow fibrosis.

The word “emperipolesis” (from the Greek word meaning “inside wandering”) was first used by Humble et al. [1] in 1956 to describe the passage of cancer cells inside megakaryocytes. Later, studies defined emperipolesis as the random passage of one cell through the cytoplasm of another with no pathological consequence for either one of them. It was then discovered that emperipolesis is particularly frequent in megakaryocytes because by transmission electron microscopy, up to 5% of those present in a normal bone marrow section are engulfed with other cell types, including red blood cells and neutrophils [2]. Additional studies described that the frequency of neutrophil emperipolesis within the megakaryocytes in the bone marrow increases under conditions of stress, such as extreme thrombocytosis [3]; during the recovery of mice from sublethal irradiation [4]; in rats with aging [5]; and in the presence of altered megakaryocyte maturation induced by either gunmetal mutation [6] or *GATA1* Arg216Gln mutation, which is one of the mutations responsible for the gray platelet syndrome [7,8]. The presence of high levels of pathological emperipolesis between megakaryocytes and neutrophils in the bone marrow of patients with myelofibrosis (MF), the most severe of the Philadelphia-negative myeloproliferative neoplasms, was first described by Schmitt et al. [9,10]. A following study from our laboratory using the *Gata1*^low^ mouse model identified that in MF, the emperipolesis between neutrophils and megakaryocytes is pathological and involves fusion of the plasma membrane of the neutrophil with that of the demarcation membrane system (DMS) of the megakaryocytes and release of the content of the neutrophil granules, such as Myeloperoxidase (MPO), in the megakaryocyte cytoplasm [11]. This process results in the death of both cells by para-apoptosis and release of the proinflammatory cytokine transforming growth factor (TGF)-*β* produced by the megakaryocytes in the microenvironment [11]. By increasing the microenvironmental bioavailability of TGF-*β*, a proinflammatory cytokine known to trigger fibrosis in numerous pathological conditions [12], emperipolesis was first hypothesized by us as an important driver for the fibrosis observed in the bone marrow of patients with MF [13].

Although the pathobiological relevance of emperipolesis in the development of MF is now well recognized [14], studies on the mechanisms that drive this process in MF are scanty. The reason for the paucity of these studies is the fact that robust detection of emperipolesis relays on observations by transmission electron microscopy, a cumbersome technique that is not commonly available. In fact, because the diameter of a megakaryocyte and a neutrophil is 80 *μ*m and 10−20 *μ*m, respectively, the thickness of a section used for standard optical microscopy (3 *μ*m) does not allow to unequivocally distinguish colocalization from emperipolesis between two cell types. The recent development of confocal microscopy, which allows three-dimensional reconstructions of cells, has finally provided a user-friendly tool to determine the events occurring inside cells. A recent study has coupled transmission electron microscopy and time-lapse observations by confocal microscopy to detail the emperipolesis occurring between normal neutrophils and normal megakaryocytes [15]. This process may occur in two very different fashions. It can be fast (<10 minutes), with the neutrophils assuming an amoeboid morphology as they quickly pass through the megakaryocyte. This fast emperipolesis likely corresponds to that described by Humble et al. [1]. However, there is also a slow emperipolesis (>60 minutes), during which neutrophils are retained inside the megakaryocyte cytoplasm for a long time and localize themselves near the nucleus. The function of this slow process is unknown. However, in both processes, the cell membranes of the megakaryocytes and neutrophils remain intact.

Inspired by this recent study, we revisited the pathological emperipolesis occurring in MF. We first developed a user-friendly and robust method to evaluate the frequency of emperipolesis between megakaryocytes and neutrophils in patients with myeloproliferative disorders and in the *Gata1*^low^ mouse model. Then, we evaluated whether treatment of *Gata1*^low^ mice with reparixin, a noncompetitive allosteric inhibitor of the CXCR1/2 signaling [16,17] that reduced fibrosis in this model [18], had any effect on the levels of emperipolesis between neutrophils and megakaryocytes. The rationale for this experiment is provided by the notion that the proinflammatory cytokine interleukin 8 (IL-8), the ligand for CXCR1/2, expressed at supranormal levels by the megakaryocytes from patients with MF [19,20] and mouse models [21,22], is a driver for neutrophil chemotaxis [23], a process that precedes the actual emperipolesis between these cells and the megakaryocytes. We hypothesized that inhibition of CXCR1/2, by reducing chemotaxis, would also reduce emperipolesis.

## METHODS

### Patients

Consecutive sections from bone marrow biopsy samples of subjects with a confirmed diagnosis of MF (three patients) or post−essential thrombocythemia (ET) (three patients) using World Health Organization (WHO) criteria were provided as deidentified material by the Center of Research and Innovation of University Hospital Careggi in Florence, Italy. Three patients with early-stage follicular lymphoma and no fibrosis were included in the study as controls. The clinical information on the subjects is summarized in Table 1. The study was approved by the local institutional review board (#14560, 2019, Mynerva project), and the patients provided informed written consent, according to the Declaration of Helsinki for human rights.

### Mice

*Gata1*^low^ mice were generated at the Animal Facility of Istituto Superiore di Sanità and genotyped at birth as described [24]. Mice that did not carry the mutation were used as wild-type (WT). The effect of reparixin on emperipolesis was studied on samples stored in the tissue bank from the mice treated with reparixin for 20 days, as described in the study by Verachi t al. [18]. All the experiments were performed according to the protocols D9997.121 approved by the Italian Ministry of Health on September 2, 2021 and according to the European Directive 86/609/EEC.

### Confocal Microscopy

Paraffin-embedded tissues were cut into consecutive 3-*μ*m sections, dewaxed in xylene, rehydrate and treated with citrate buffer (pH = 6) for 30 minutes at 90°C for antigen retrieval. Sections were incubated with appropriate primary anti-CD42b (Ab183345, rabbit monoclonal, Abcam, and SC-271171, mouse monoclonal, Santa Cruz Biotecnology) and either anti-Ly6B (Ab25377, rat monoclonal, Abcam) or anti−neutrophil elastase (PA5–115648, rabbit polyclonal, Invitrogen) for 2 hours at room temperature. For each case, secondary Alexa Fluor 488 and/or Alexa Fluor 568-conjugated donkey anti-rabbit, anti-rat, or anti-mouse (both from Invitrogen) were added for 1 hour. Nuclear counterstaining was performed using Hoechst 33342, trihydrochloride, trihydrate (Invitrogen), and the samples were mounted with Fluor-shield histology mounting medium (Catalog F6182–10MG, Sigma-Aldrich). Fluorescent images were collected with a Nikon A1 confocal laser microscope (Nikon), acquired with Imaging Software NIS-Elements (Nikon), and processed with Fiji software (US National Institutes of Health). Events were quantified by examination of images acquired at × 60 magnification from at least 10 randomly selected areas per section. The total area analyzed for each sample was 0.448 mm^2^.

### Statistical Analysis

Data were analyzed and plotted using GraphPad Prism 8.0.2 software (GraphPad Software) and presented as mean (±SD). Comparisons between the two groups were performed with 1-way (analysis of variance) ANOVA, whereas comparisons between multiple groups were performed with Tukey’s multiple comparisons test. Differences were considered statistically significant at a *p* value of <0.05.

## RESULTS

The bone marrow from patients with MF contains great numbers of both megakaryocytes and neutrophils that are preferentially located around or emperipolesed with megakaryocytes.

**Figure 1:**
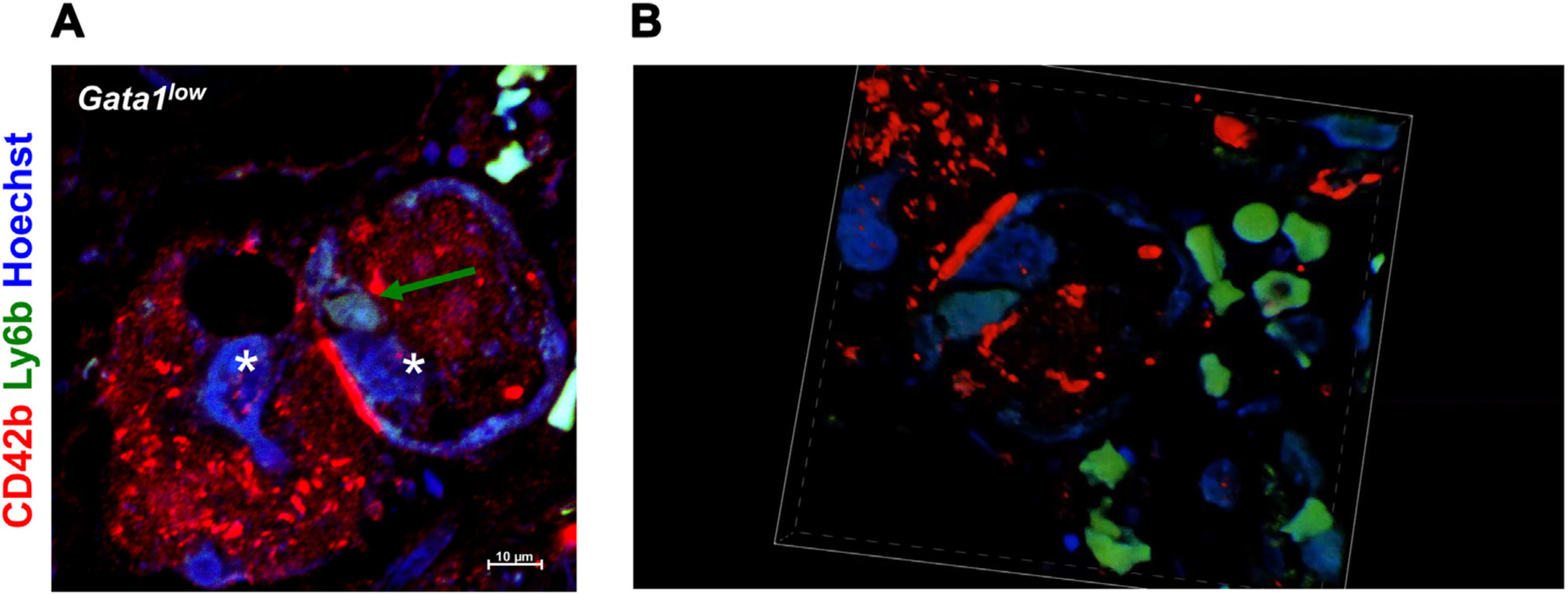
Validation of confocal microscopy observations with antibodies specific for the megakaryocytes and the neutrophils as a method to detect emperipolesis in the bone marrow. **(A)** A representative image acquired by confocal microscopy of bone marrow sections from a *Gata1*^low^ mouse stained with CD42b (red, megakaryocyte) and Ly6b (green, neutrophil). Nuclei are counterstained with Hoechst (blue). The asterisks indicate two representative megakaryocytes (in red), one of which contains Ly6b-positive cells (blue, indicated by the arrow). This image is from an area of the bone marrow parenchyma close to a vessel, as indicated by the presence of red blood cells (the autofluorescent green Hoechst-negative cells). **(B)** Three-dimensional reconstruction of the emperipolesed megakaryocyte shown in Figure 1A showing that the Ly6b signal is inside the cytoplasm of the CD42b-positive cells (Supplementary Video E1). Original magnification, 60 ×.

**Figure 2:**
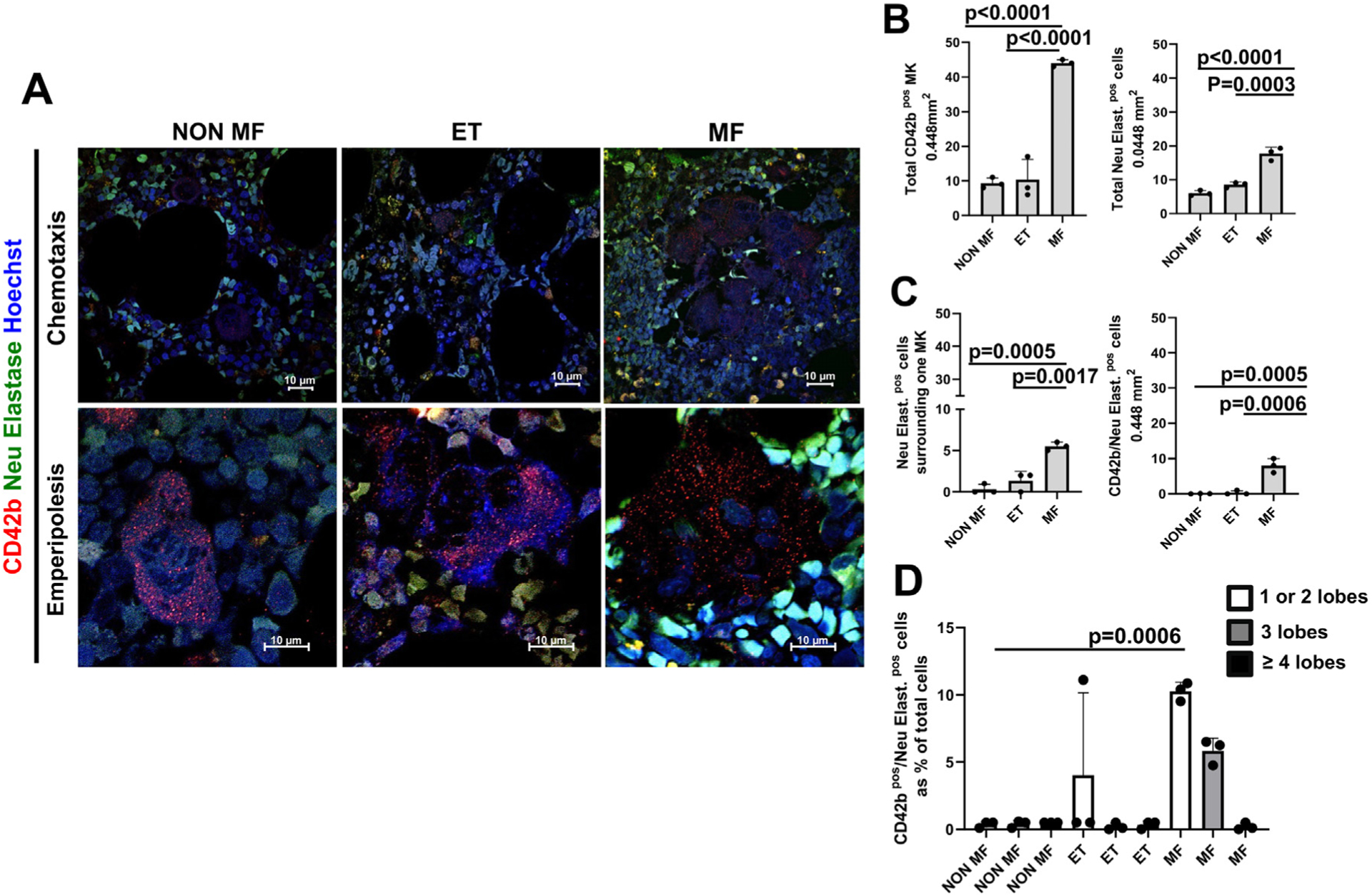
The bone marrow from patients with MF contains significantly greater numbers of megakaryocytes (MKs) (CD42b-positive [CD42b^pos^] cells, red) and neutrophils (neutrophil elastase−positive [Neu Elast.^pos^] cells, green), which are located around or emperipolesed with MKs, than the bone marrow from patients without MF or with ET. **(A)** Confocal microscopy analyses of bone marrow sections from one patient without MF, one patient with ET, and one patient with MF stained with CD42b (red), neutrophil elastase (green), and Hoechst (blue). The panels on the top and bottom present representative images showing the frequency of neutrophils around (as a surrogate marker of chemotaxis) and inside (as evidence of emperipolesis) the MKs, respectively. Original magnification, 60 × . **(B)** Frequency of MKs (CD42b-positive cells) and neutrophils (neutrophil elastase−positive cells) in bone marrow sections from three patients without MF, three patients with ET, and three patients with MF. **(C)** Frequency of neutrophils (neutrophil elastase−positive cells) surrounding or emperipolesed with MKs (CD42b-positive cells) of the different groups. **(D)** The number of nuclear lobules (as detected by Hoechst staining) contained in the cytoplasm of MKs emperipolesed by neutrophils present in bone marrow sections from the patients. MKs were divided into cells containing 1−2, 3, or >4 lobules. Data are presented as mean (±SD) and as values for individual patients (each dot represents a different patient). In Figure 2B−D, data are presented as mean (±SD) and as values per individual patient (each dot represents a different patient). *p* values were calculated by Tukey’s multiple comparisons test.

The power of confocal microscopy, which allows us to observe cells along all the thickness of a section, coupled with the specificity of the CD42b (megakaryocytes) and Ly6b/neutrophil elastase antibodies (neutrophils) allows determinations of the emperipolesis events occurring between megakaryocytes and neutrophils as robust as those previously obtained by transmission electron microscopy (Figures 1 and 2). Because the Ly6b antibody (Ab25377) recognizes both neutrophils and monocytes, in sections stained with this antibody, neutrophils were positively identified by morphology as well on the basis of the kidney shape of their nuclei by Hoechst. For precision, in future studies, neutrophils could be univocally recognized with the Ly6B.2 antibody (Ab53457).

Using this technical advantage as a foundation, we confirmed that the bone marrow from patients with MF contains five times more megakaryocytes than that from patients with ET or control patients (non-MF) (Figure 2A, B). This study also indicated that the bone marrow from these patients contains a greater number of neutrophils (20 vs. 5 per 0.448 mm^2^, in MF vs. non-MF or ET, respectively) (Figure 2B) mostly localized around megakaryocytes (on average, five neutrophilsin close contact to each megakaryocytes) (Figure 2C). In addition, a great number (25%) of the MF megakaryocytes were emperipolesed by neutrophils (10 megakaryocytes in emperipolesis over a total of 40 megakaryocytes per area) (Figure 2C).

The process of terminal megakaryocyte maturation involves a series of endomitosis, which progressively increases the DNA content of the cells from 2 N up to 64−128 N. To accommodate its increased DNA content, the size of the nucleus of the megakaryocytes increases with maturation and acquires a multilobated morphology [25]. Although much less precise than flow cytometric determinations, the number of nuclear lobes may be used as a surrogate marker for the DNA content and maturation state of megakaryocytes. To assess whether the emperipolesis involved a specific state of megakaryocyte maturation, we determined the number of nuclear lobes present in cells in emperipolesis with neutrophils (Figure 2D). This study revealed that most of the megakaryocytes engaged in emperipolesis have a low number of nuclear lobes, an indication that these cells are immature.

The bone marrow from *Gata1*^low^ mice contains great numbers of both megakaryocytes and neutrophils. In addition, in this case, the neutrophils are preferentially located around or emperipolesed with megakaryocytes.

**Figure 3:**
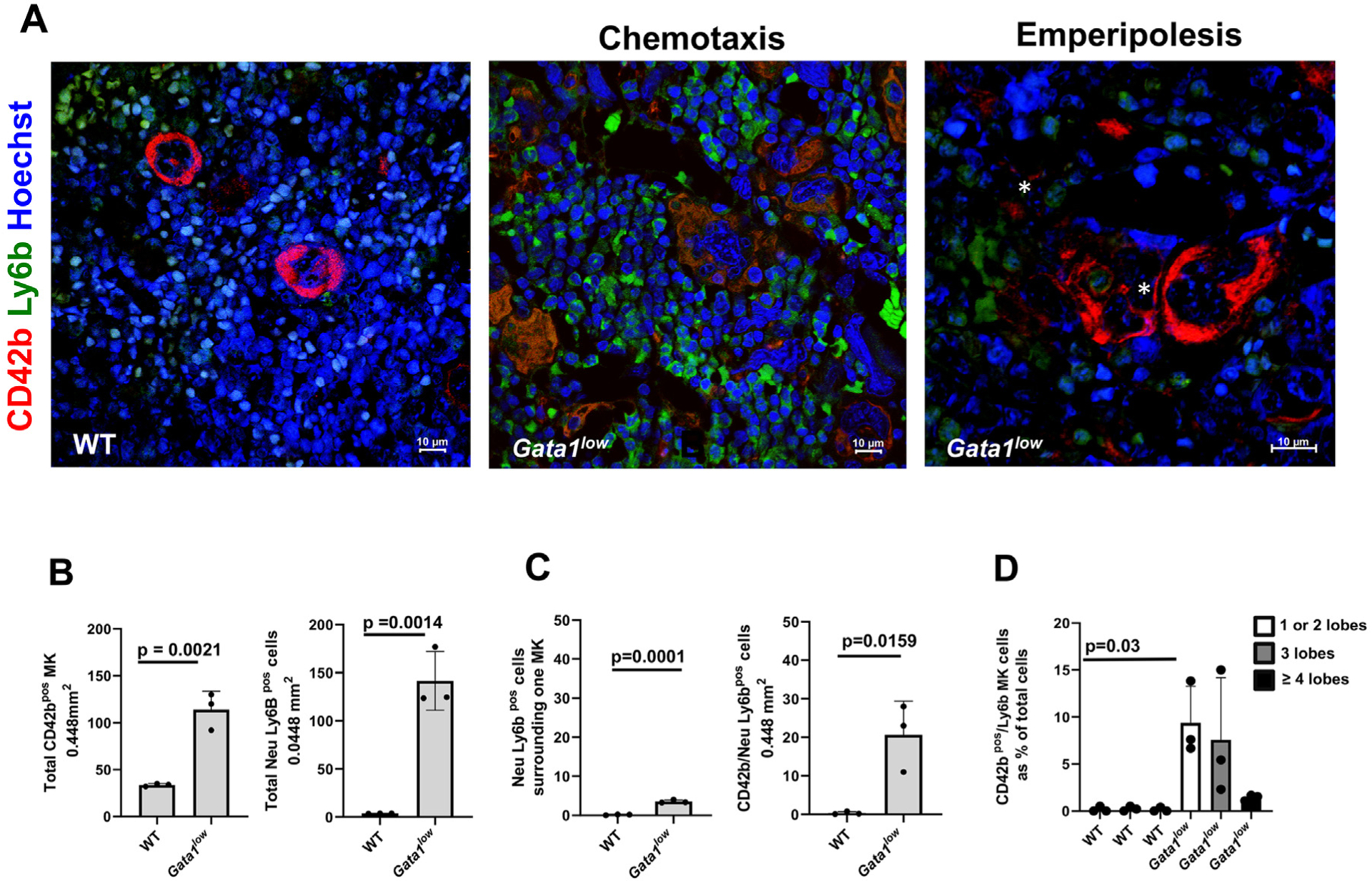
The bone marrow from *Gata1*^low^ mice contains significant greater numbers of megakaryocytes (MKs) (CD42b-positive [CD42b^pos^] cells, red) and neutrophils (Ly6b-positive [Ly6b^pos^] cells, green), which are located around or emperipolesed with MKs, than the bone marrow from WT littermates. **(A)** Confocal microscopy analyses of bone marrow sections from one *Gata1*^low^ mouse (8 months old) and one age-matched WT littermate, as indicated, stained with CD42b (red), Ly6b (green), and Hoechst (blue). The panels for *Gata1*^low^ mice show a group of MKs surrounded by neutrophils (as a surrogate marker of chemotaxis) and one MK in emperipolesis with neutrophils (asterisk in the right panel). Original magnification, 60 × . **(B)** Frequency of MKs (CD42b-positive cells) and of neutrophils (Ly6b-positive cells) in bone marrow sections from three WT and three *Gata1*^low^ mice. **(C)** Frequency of neutrophils (Ly6b-positive cells) surrounding or emperipolesed with MKs (CD42b-positive cells) in the two groups. **(D)** The number of nuclear lobules (as detected by Hoechst staining) contained in the cytoplasm of MKs in emperipolesis, with the neutrophils present in bone marrow sections from the two groups. MKs were divided into cells containing 1−2, 3, or >4 lobules. Data are presented as mean (±SD) and as values for individual patients (each dot represents a different patient). In Figure 3B−D, data are presented as mean (±SD) and as values per individual patient (each dot represents a different patient). *p* values were calculated by one-way ANOVA in Figure 3B and C and by Tukey’s multiple comparisons test in Figure 3D.

To confirm the results previously published by our group [11], we performed confocal microscopy observations with the CD42b (megakaryocytes) and Ly6b (neutrophils) antibodies of the bone marrow sections from *Gata1*^low^ mice and age-matched WT littermates (Figure 3). The mice were 8 months old when they expressed the MF phenotype [24]. This analysis indicated that the bone marrow from *Gata1*^low^ mice not only contains 3−4 times more megakaryocytes than that from the age-matched littermates but also contains >10 times more neutrophils (Figure 3A, B). Some of the neutrophils were localized around or emperipolesed with the megakaryocytes (Figure 3C). In addition, in the case of *Gata1*^low^ mice, most of the megakaryocytes in emperipolesis had a low number of nuclear lobes (Figure 3D).

Treatment with reparixin does not affect the frequency of megakaryocytes but drastically reduces the frequency of neutrophils and the number of neutrophils around or emperipolesed with megakaryocytes.

**Figure 4:**
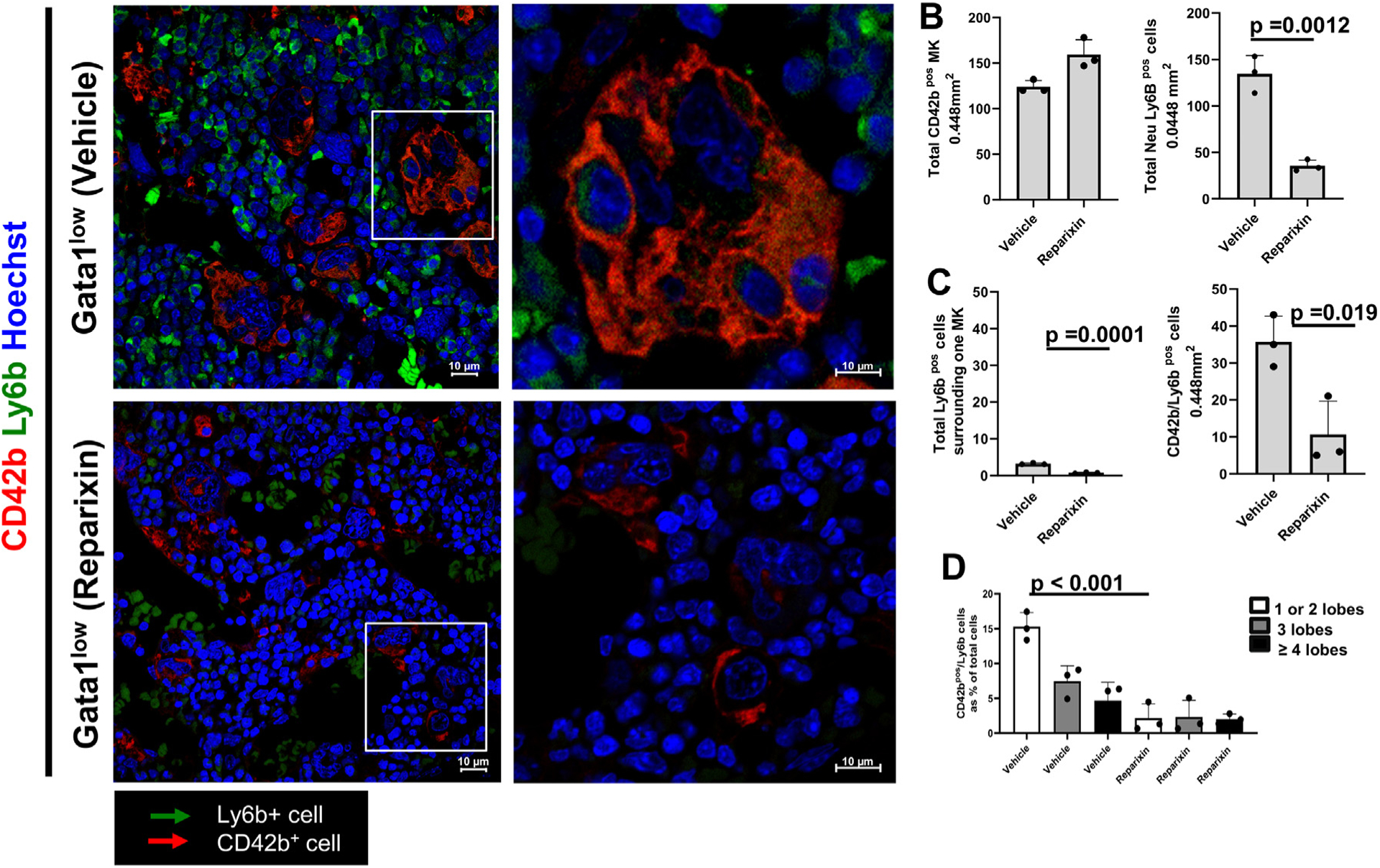
Treatments with reparixin do not affect the frequency of megakaryocytes (MKs) present in the bone marrow but significantly decreases that of neutrophils (Ly6b-positive [Ly6b^pos^] cells, green). It also decreases the frequency of neutrophils around or emperipolesed with MKs in the bone marrow of *Gata1*^low^ mice. **(A)** Confocal microscopy analyses of bone marrow sections from one *Gata1*^low^ mouse (9 months old) treated either with a vehicle or with reparixin, as indicated, stained with CD42b (red), Ly6b (green), and Hoechst (blue). The rectangle in the panels on the left are shown at greater magnification on the right. Representative MKs and neutrophils are indicated by the red and green arrows, respectively. Original magnification, 60 × . **(B)** Frequency of MKs (CD42b-positive [CD42b^pos^] cells) and neutrophils (Ly6b-positive cells) in bone marrow sections from three WT and three *Gata1*^low^ mice. **(C)** Frequency of neutrophils (Ly6b-positive cells) surrounding or emperipolesed with MKs (CD42b-positive cells) in the two groups. **(D)** The number of nuclear lobules (as detected by Hoechst staining) contained in the cytoplasm of MKs in emperipolesis with the neutrophils present in bone marrow sections from the two groups. MKs were divided into cells containing 1−2, 3, or >4 lobules. Data are presented as mean (±SD) and as values for individual patients (each dot represents a different patient). In Figure 4B−D, data are presented as mean (±SD) and as values per individual patient (each dot represents a different patient). *p* values were calculated by one-way ANOVA in Figure 4B and C and by Tukey’s multiple comparisons test in Figure 4D.

Because chemotaxis, a process induced by CXCL1/IL-8 [20], must precede the emperipolesis between two cells, we determined whether treatment with the CXCL1 inhibitor reparixin for 20 days affected the emperipolesis between neutrophils and megakaryocytes in the bone marrow from *Gata1*^low^ mice (Figure 4). Treatment with reparixin had no effect on the frequency of megakaryocytes in these mice, which remained similar to that observed in animals treated with vehicle (Figure 4A, B). However, it greatly decreased (by fivefold) the frequency of neutrophils, neutrophils surrounding the megakaryocytes, and neutrophils emperipolesed with them (Figure 4B, C). The reductions in emperipolesis were mostly observed in the megakaryocytes with a low number of nuclear lobes (Figure 4C).

## DISCUSSION

TGF-*β* and IL-8 are two of the proinflammatory cytokines expressed at high levels in the microenvironment of patients with MF, which are believed to be responsible for the etiopathogenesis of the disease [14]. Although the increases in the plasma levels of TGF-*β* in patients with MF and in mouse models are modest (twofold) [26], the bioavailability of TGF-*β* in the bone marrow microenvironment is extremely high and is believed to be responsible for the activation of the cells of the microenvironment promoting fibrosis [13]. By contrast, although high levels of IL-8 are detected both in the plasma, where they negatively correlate with disease progression, and in the bone marrow of these patients [19,20], the pathobiological mechanisms linking IL-8 to fibrosis have not been clearly established as yet. In a previous study, we validated the causative effect of IL-8 in the development of MF by demonstrating that treatment with the CXCR1/CXCR2 inhibitor reparixin greatly reduces in a dose-dependent fashion the levels of fibrosis present in the bone marrow from *Gata1*^low^ mice [18]. Mechanistically, in this study, reduction of fibrosis was associated with reduced microenvironmental bioavailability of TGF-*β* (by 40% at day 20) and increased content of GATA1 in megakaryocytes [18], the transcription factor necessary for their maturation [27]. The link between the inhibition of CXCL1, the murine equivalent of human IL-8, and the reduced TGF-*β* bioavailability observed in this study remained poorly defined. In addition, the consequence of the increased content of GATA1 in megakaryocytes was not clear because the treatment did not increase platelet counts, an indication that megakaryocytes had remained overall immature.

In this study, by revisiting the emperipolesis between megakaryocytes and neutrophils in MF, we provide a link between IL-8 and TGF-*β* in the pathogenesis of the disease. We first confirm that the bone marrow from patients with MF and *Gata1*^low^ mice contains great numbers not only of megakaryocytes but also of neutrophils. We also identified that in both patients and mouse models, a great number of neutrophils are located around the megakaryocytes and/or emperipolesed with them. Because IL-8 is a driver of neutrophil chemotaxis, which is a prerequisite for emperipolesis to occur [23], our data suggest that the IL-8 produced by the malignant megakaryocytes is responsible for attracting the neutrophil, triggering the emperipolesis event. It is also possible that IL-8−induced chemotaxis is responsible for the fusion between the membranes of the two cells, which mediate the release of TGF-*β* in the cytoplasm. The reduced levels of emperipolesis observed in mice treated with reparixin support this hypothesis.

Our study has many limitations. The premise that the IL-8 produced by megakaryocytes is responsible for neutrophil chemotaxis and triggering the emperipolesis process has not been formally proved. In addition, although our data indicate that “immature malignant megakaryocyte” responsible for emperipolesis may represent a subpopulation with distinctive biological functions, the identity of these cells and how their functions may be affected by the content of GATA1 have also not been investigated. These questions will be the subject of additional investigation.

Despite these limitations, these data finally identify the mechanistic link between increased IL-8 content in the plasma and increased TGF-*β* bioavailability in the bone marrow. This link provides a rationale to explain why greater levels of IL-8 represent a biomarker for adverse disease progression: in other words, the greater the IL-8, the greater the emperipolesis and TGF-*β* release in the microenvironment and the higher the levels of fibrosis. Given the impracticality of measuring TGF-*β* bioavailability and fibrosis in bone marrow biopsy samples from patients with MF, we suggest the plasma levels of IL-8 as a biomarker for levels of fibrosis.

## Supplementary Material

MMC1

## Figures and Tables

**Table 1:** Clinical information on the patients enroleld in the study

	Age (y)	Sex	Driver mutation	Platelets (10^9^/L)	Bone marrow fibrosis grade	Spleen size (cm by ecoscan)
No MF						
1	65	Male	Lymphoma	NA^a^	0	12
2	46	Female	Lymphoma	NA	0	11
3	57	Male	Lymphoma	NA	0	22
ET						
4	27	Male	CALR type 1	1017	0	10
5	53	Female	CALR type 1	824	0	10
6	34	Female	CALR type 1	929	0	9
MF						
7	80	Female	JAK2 (V617F)	1296	0	15
8	65	Female	CALR type 2	509	1	20
9	63	Female	JAK2 (V617F)	12	2	30

*NA* ^a^=Not available but supposedly within normal ranges.

## Data Availability

The individual data are available on request. The original confocal microscopy images of all the individual samples described in the manuscript are available upon request to the corresponding author.

## HIGHLIGHTS

- Myelofibrosis is characterized by high numbers of neutrophils and MKs in the bone marrow.
- Neutrophils are localized either around or in emperipolesis with the MKs.
- The CXCL1inhibitor reparixin reduced emperipolesis and fibrosis in animal models.
